# Co-Evolution of Breast Milk Lipid Signaling and Thermogenic Adipose Tissue

**DOI:** 10.3390/biom11111705

**Published:** 2021-11-16

**Authors:** Tamás Röszer

**Affiliations:** Institute of Neurobiology, Faculty of Science, Ulm University, 89081 Ulm, Germany; tamas.roeszer@uni-ulm.de

**Keywords:** metabolism, neonate care, breastfeeding, beige adipose tissue, obesity

## Abstract

Breastfeeding is a unique and defining behavior of mammals and has a fundamental role in nourishing offspring by supplying a lipid-rich product that is utilized to generate heat and metabolic fuel. Heat generation from lipids is a feature of newborn mammals and is mediated by the uncoupling of mitochondrial respiration in specific fat depots. Breastfeeding and thermogenic adipose tissue have a shared evolutionary history: both have evolved in the course of homeothermy evolution; breastfeeding mammals are termed “thermolipials”, meaning “animals with warm fat”. Beyond its heat-producing capacity, thermogenic adipose tissue is also necessary for proper lipid metabolism and determines adiposity in offspring. Recent advances have demonstrated that lipid metabolism in infants is orchestrated by breast milk lipid signals, which establish mother-to-child signaling and control metabolic development in the infant. Breastfeeding rates are declining worldwide, and are paralleled by an alarming increase in childhood obesity, which at least in part may have its roots in the impaired metabolic control by breast milk lipid signals.

## 1. The Lipid-Rich Breast Milk Is a Key Energy Source in Newborn Mammals

**Figure 1:**
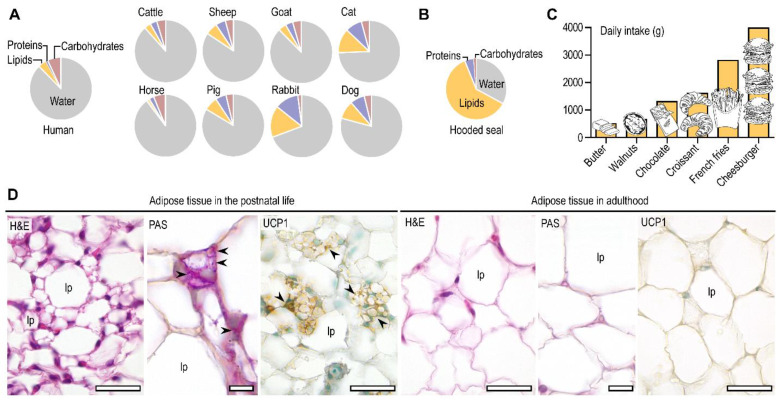
Fat intake and adipose tissue during postnatal life. (**A**) Composition of the breast milk in humans and various mammals. (**B**) Composition of breast milk in the hooded seal *Cystophora cristata*, which is recognized as the most fat-rich breast milk among mammals [20]. (**C**) Illustration of the daily fat intake of a breastfed newborn human infant extrapolated to a human adult. The graph shows the weight of various food items which provide the daily fat intake of a breastfed infant (e.g., fat content of ~1250g chocolate is equivalent with the daily fat intake of a breastfed infant). (**D**) Histology of the subcutaneous adipose tissue after birth and in adulthood in the mouse [17]. Notably, the infant adipose tissue is rich in multilocular (“beige”) adipocytes, contains PAS^+^ glycogen granules, and expresses UCP1 protein. These traits allow an active lipid catabolism, lipid synthesis from glycogen, and heat generation from fatty acid oxidation. In turn, the adult adipose tissue is built up from unilocular adipocytes, which do not store glycogen, nor express UCP1. Postnatal development of the subcutaneous adipose tissue is associated with a shift from lipid catabolism and thermogenesis to lipid storage and thermal insulation [17]. H&E, hematoxylin and eosin; PAS, periodic acid Schiff (staining glycogen); UCP1, uncoupling protein 1 (immunohistochemistry); lp, lipid droplet. Arrowheads label glycogen granules and UCP1^+^ cell clusters. Scale bar 50 μm.

The principle energy source of suckling mammals (including humans) is fat, which is obtained from lipid-rich breastmilk (Figure 1A,B) [1,2]. Dietary lipids are potent signals which control lipid metabolism and the development and functioning of the fat-storing adipose tissue [3,4,5]. They play specific roles in adipose tissue physiology (for instance, by activating lipid sensing nuclear receptors or by giving rise to inflammatory or pro-resolving lipid mediators) [6,7,8,9]. However, they may also trigger obesity-associated diseases such as adipose tissue inflammation, insulin resistance, and diabetes [3,10,11]. The effects of dietary lipids are mostly studied in the context of obesity in adulthood, and we know much less about the signaling role of dietary lipids after birth.

This paucity of information is somewhat surprising, since the early postnatal life is the peak of dietary fat intake. To illustrate this, extrapolating the average daily fat intake of a breastfed infant to an adult would result in a non-physiological and a hardly-possible degree of fat consumption (Figure 1C) [12,13,14,15,16]. Moreover, newborns are capable of heat generation from lipids, a trait which declines by adulthood (Figure 1D), especially in obese individuals [17]. Restoring this ability of the adipocyte is considered today as a possible mechanism to reduce obesity [18]. Recent advances have demonstrated that some breast milk lipid species establish a mother-to-child signaling, promoting heat generation from fat and protecting from obesity [12,19].

Worryingly, the incidence of obesity, insulin resistance, and diabetes is increasing among children and young adults [21], and is considered one of the most serious public health challenges of the 21st century [22]. Prevalence of overweight individuals doubled among children and tripled in teenagers between 1980 and 2000 [23]; in 2010, approximately 6.7% of preschool-aged children were overweight or obese worldwide [24], and in 2016, the number of overweight children under the age of five was estimated to be over 41 million globally. This number is predicted to rise further [22]. Increased body fat increases the preference for high-fat food in human [25], and the likelihood of successful long-term weight loss in obese young adults is as low as 10–20% [26], showing that early life obesity is a likely root of life-long obesity.

Infancy is one of the critical periods which determine obesity in adulthood [27]. Infants whose body weight exceeded the 90th percentile at three to six months of age have an increased risk of obesity when they reach 20–30 years of age [28]. Similarly, increased rate of body weight gain [29] and being overweight at the first year of life increase one’s probability of obesity as a young adult [30,31]. Increased adiposity in early childhood (before 5.5 years of age) is a predictor of obesity and obesity-associated diseases in adulthood [31,32,33,34,35,36]. Following infancy, adiposity declines in humans, and at the age of 5–7 years a rebound of adipose tissue expansion happens as a physiological process [32]. However, when adiposity rebound appears earlier due to childhood obesity, it leads to obesity in adulthood [32]. In primates, for instance, excess calorie intake during infancy leads to obesity at the period of sexual maturation [37], when a rebound of adipose tissue expansion happens [38]. These findings show the impact of infant nutrition in the development of adult obesity [35,36].

Infancy is one of the critical periods which determine obesity in adulthood [27]. Infants whose body weight exceeded the 90th percentile at three to six months of age have an increased risk of obesity when they reach 20–30 years of age [28]. Similarly, increased rate of body weight gain [29] and being overweight at the first year of life increase one’s probability of obesity as a young adult [30,31]. Increased adiposity in early childhood (before 5.5 years of age) is a predictor of obesity and obesity-associated diseases in adulthood [31,32,33,34,35,36]. Following infancy, adiposity declines in humans, and at the age of 5–7 years a rebound of adipose tissue expansion happens as a physiological process [32]. However, when adiposity rebound appears earlier due to childhood obesity, it leads to obesity in adulthood [32]. In primates, for instance, excess calorie intake during infancy leads to obesity at the period of sexual maturation [37], when a rebound of adipose tissue expansion happens [38]. These findings show the impact of infant nutrition in the development of adult obesity [35,36].

Infancy is one of the critical periods which determine obesity in adulthood [27]. Infants whose body weight exceeded the 90th percentile at three to six months of age have an increased risk of obesity when they reach 20–30 years of age [28]. Similarly, increased rate of body weight gain [29] and being overweight at the first year of life increase one’s probability of obesity as a young adult [30,31]. Increased adiposity in early childhood (before 5.5 years of age) is a predictor of obesity and obesity-associated diseases in adulthood [31,32,33,34,35,36]. Following infancy, adiposity declines in humans, and at the age of 5–7 years a rebound of adipose tissue expansion happens as a physiological process [32]. However, when adiposity rebound appears earlier due to childhood obesity, it leads to obesity in adulthood [32]. In primates, for instance, excess calorie intake during infancy leads to obesity at the period of sexual maturation [37], when a rebound of adipose tissue expansion happens [38]. These findings show the impact of infant nutrition in the development of adult obesity [35,36].

Notably, breast-fed children have a lower probability to develop obesity and diabetes later in life [39,40,41,42,43,44,45,46]; breastfeeding controls the rate of weight gain and adiposity rebound [47], artificially reared mice develop obesity before reaching sexual maturity [12], and infant formula feeding induces diabetes in diabetes-prone rats [48]. Moreover, the rapid global increase of childhood obesity incidence is paralleled with the decline of breastfeeding rate today [35].

There are various mechanisms that explain the metabolic benefits of breastfeeding. For instance, breast milk establishes the normal flora in the infant [49], which is eventually necessary for thermogenic fat differentiation [50] and protects from autoimmune diabetes [49]. Breastfeeding also affects the development of food preference, and breastfed infants have a higher acceptance of new types of food [51] and retain a healthier dietary pattern later in life [52]. Moreover, the impact of signaling through breast milk lipids is a novel mechanism which allows breastfeeding to control adipose tissue development in infancy. This review shows that breast milk lipid signals have the potential to increase heat generation (and the “burning off” of excess calories) from fat. Both breastfeeding and thermogenic potential of the adipose tissue have evolved in the quest for maintaining stable core body temperature, suggesting a shared evolutionary history of the two traits and a possible link between breastfeeding and thermogenic fat differentiation.

## 2. Fat Is a Relevant Metabolic Fuel after Birth

During intrauterine life, the fetus is supplied by the maternal metabolism, which provides a carbohydrate-rich fuel. In humans, the adipose tissue primordia begins its expansion during the latter part of gestation, and maternal ketone bodies and glucose constitute the main lipogenic substrates for the fetus [1]. Approximately 70% of fetal glucose is converted into fat before birth [53,54], and primordial fat cells contain glycogen and neutral lipids [17,55,56,57]. At birth, these energy reserves account for 1% and 16% of the body weight, respectively [2].

Extrauterine existence is marked by a rapid shift from a carbohydrate-based metabolism to one mainly of lipids, concomitant with an increase in lipolysis and the release of glycerol and free fatty acid from fat cells [1,2]. Glycerol is converted to glucose, free fatty acids are oxidized or re-esterified in the adipose tissue [58]. Breast milk is rich in lipids, of which 85–90% are absorbed and metabolized by a term infant [54]. The plasma lipid profile of a breastfed infant thus reflects the lipid composition of the breast milk [59], and also the maternal adipose tissue and plasma lipids [45,60]. Indeed, lipid digestion begins in the buccal cavity in the newborn, owing to the presence of lingual lipase in the mouth and lipases of breast milk [61].

Mammals are homeothermic animals and the fetus develops in a thermally stable environment. The scenario changes at birth, with a large demand of energy to sustain core body temperature in a hypothermic environment [62], and parental care (i.e., swaddling or wrapping) is necessary to protect the infant from hypothermia [63]. This is largely maintained by non-shivering thermogenesis, which utilizes the uncoupling of mitochondrial oxidative respiration to generate heat [62]. It is estimated that the heat produced in a human term infant is generated mostly by fat (64%) and carbohydrate (28%) metabolism, and only 4% by protein catabolism [2,64].

Overall, the relative metabolic importance of fat is approximately three times greater in a newborn than in an adult human [64]. While the rate of fatty acid synthesis per unit wet weight is higher in infancy than in adulthood [65], lipids are rapidly catabolized to generate energy and heat by virtue of the unique metabolic profile of the adipose tissue in the newborn.

## 3. Dual Metabolic Roles of the Newborn Adipose Tissue: Fatty Acid Oxidation and Lipogenesis

**Figure 2:**
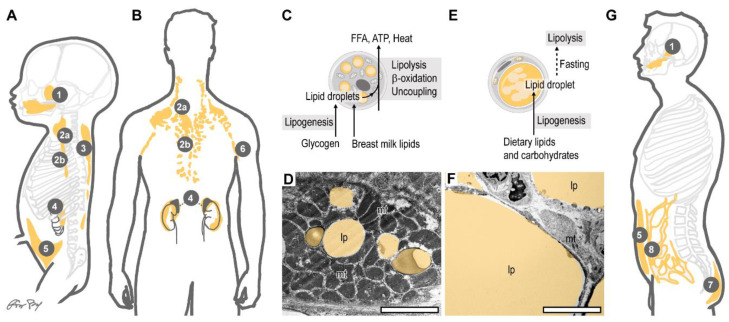
Thermogenic and fat storing adipose tissue depots. (**A**) Thermogenic fat depots of a human term infant. (**B**) Thermogenic fat depots of a human adult. 1: buccal fat pad, 2a: supraclavicular fat depot, 2b: mediastinal fat depot, 3: interscapular and subcutaneous fat of the trunk, 4: fatty capsule of the kidney, 5: abdominal and inguinal subcutaneous fat depot, 6: axillary fat depot, 7: gluteal fat pad, 8: omental fat (**C**) Fat metabolism of the adipose tissue in a newborn. Release of free fatty acids (FFA), ATP and heat generation are the key tasks of the newborn fat. The infant adipocytes also generate lipids from stored glycogen [69]. Accordingly, the subcutaneous adipose tissue contains 1 mg/g glycogen in the newborn human, which drops to 0.3 mg/g within 120 h after birth [74]. The adult adipose tissue contains 0.25 mg/g glycogen in human [64]. (**D**) Transmission electron microscopy image of an adipocyte from the inguinal region of a human newborn. Lipid droplets (lp) are secondarily colored for better visibility. Note the abundance of mitochondria (mt). Scale bar 2 μm. Samples from our previous study [12]. (**E**) Fat metabolism of the adult adipose tissue. Lipogenesis and the storage of neutral lipids is the major function of the fat cells. Stored fat serves as energy reserve for periods of fasting, and also serves as thermal insulation. (**F**) Transmission electron microscopy image of an adipocyte from the inguinal fat depot of an adult human [12]. Note the scarcity of mitochondria (mt) and abundance of lipids (lp). Scale bar 2 μm. (**G**) Fat storing adipose tissue depots of a human adult. Some thermogenic depots persist throughout life (supraclavicular, axillar, mediastinal fat, fatty capsule of the kidney [75,76,77,78]), while others transform into fat storing depots by adulthood (buccal fat pad, abdominal and inguinal subcutaneous fat depots [12,73]), and some expand considerably in adulthood (omental fat, gluteal fat pad [66,79]).

A four-month-old infant has 26–33% body fat, accounting for 45–50% of the total body weight [64]. Overall, 12% of the total body fat can be found in the adipose tissue in a human newborn, versus 3.3% in a normal adult [64]. The human newborn has large subcutaneous fat depots, and the omental fat (a large visceral fat depot in the adult) is lacking (Figure 2A,B). Some visceral fat depots are, however, already established in the newborn, including the fatty capsule of the kidney and fat depots around the large arteries [66]. The human infant also has specific subcutaneous fat pads, such as the buccal fat pad, which assists in suction during breastfeeding [67]. Other large fat pads are found at the ischiorectal fossa and in the eye sockets. Also, the palms have fibrous fat pads, and a fat pad can be found in the axillary region associated with the axillary fascia, which unites the fascia of the pectoral muscle, the latissimus dorsi and the brachial muscles [66].

Infant fat is richer in adipocytes than adult fat: the estimated number of adipocytes in a human infant at six months of age is 130,000/kg body weight versus 45,000/kg body weight in an adult [64]. While the degree of adiposity seems to be prominent, oxidative metabolism is greater in infant fat than in adult fat, allowing the rapid utilization of energy stored in fat. Indeed, the human newborn subcutaneous fat depot utilizes almost 4000 μmol O_2_/kg tissue, versus ~1000 μmol O_2_/kg in the adult subcutaneous fat depot [64]. Fatty acid mobilization is oxygen-dependent in the newborn adipose tissue [68] and mitochondrial enzyme levels are also two- to four-fold higher in the newborn subcutaneous fat cells than in their adult counterparts [69].

**Figure 3:**
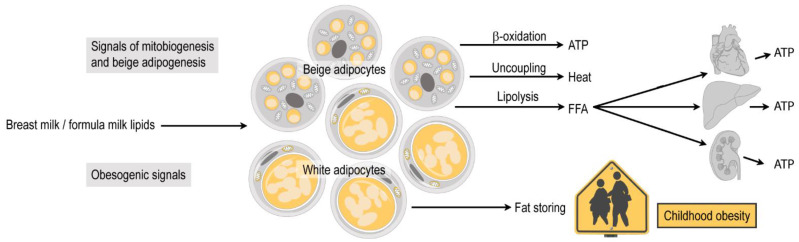
Duality of lipid metabolism in the infant subcutaneous adipose tissue. The adipose tissue in the infant has mitochondria-rich “beige” or “brown” adipocytes and fat storing “white” adipocytes. The beige or brown adipocytes actively break down neutral lipids, and oxidize fatty acids via mitochondrial β oxidat**ion to** produce ATP, or use fatty acids to generate heat from uncoupled terminal electron transport of the mitochondria. Moreover, they release free fatty acids (FFA) into the blood circulation, which are used by energy-demanding organs, such as the heart, the liver and the renal cortex to produce ATP. White adipocytes have lipogenic activity and build up lipid stores. Breast milk lipid signals sustain the differentiation of beige/brown adipocytes. When the amount of the beige/brown adipocytes decreases (for instance, due to insufficient breastfeeding or in response to obesogenic signals), childhood obesity develops.

The fatty acid oxidizing activity of the infant adipose tissue is necessary to metabolize the lipid-rich breast milk (or lipid-rich formula milk) (Figure 2C). Fatty acids are the preferred metabolic fuels of energy-demanding organs in the infant, such as the heart, the renal cortex, and the small intestine, and their oxidation in the liver remains active throughout the breastfeeding period [62] (Figure 3). In general, the ratio between fatty acid oxidation and glycolysis with respect to systemic metabolic homeostasis decreases considerably with age [64].

In addition to being a key energy source after birth, fatty acids are also fuels of heat production in the infant [62]. The newborn has to maintain body temperature, and fatty acid oxidation in the uncoupled mitochondria of adipocytes provides thermogenic capacity to the fat depots. Hypoxia is known to inhibit heat production in the newborn, underlying the importance of mitochondrial respiration for heat generation [64]. Cold exposure in the human infant increases peak oxygen uptake (VO_2_), and also increases the levels of free fatty acids and glycerol in the circulation, whereas hypoxia diminishes fatty acid mobilization and lipolysis in response to β-adrenergic stimulation [70] and cold [71,72]. These data indicate that mitochondrial respiration produces heat through lipolysis and fatty acid oxidation. Breastfeeding sustains thermogenic fat cells [12], and the loss of thermogenic fat is associated with obesity in children (reviewed in [73]).

While rapid lipolysis is evident after birth, de novo fatty acid synthesis, lipogenesis, and lipid accumulation in the adipocytes begins soon after, which is accompanied by glycogen catabolism in the primordial fat cells [56] (Figure 2C). It is plausible that this build-up of lipid stores in the adipose tissue is necessary to maintain a stable supply of fatty acids. The expansion of lipid stores continues in infancy, and is fueled by lipids consumed from breast milk or formula milk [1].

Infant adipocytes thus have a dual metabolic profile: they esterify fatty acids to build-up lipid stores, but they also actively mobilize lipids to generate heat or to release fatty acids (Figure 2C,D and Figure 3). Adult adipose tissue is, by contrast, a predominantly lipid storage site, and lipid mobilization occurs only in response to starvation (Figure 2E–G). Signaling processes that govern the metabolic profile of the infant adipose tissue hence determine whether lipids are used for energy and heat production, or are stored excessively, causing childhood obesity (Figure 3).

## 4. Infant Subcutaneous Fat: Brown, White or Beige?

Traditionally, two types of adipose tissue are recognized: the fat-storing white adipose tissue (WAT) and the fat-oxidizing/heat-producing brown adipose tissue (BAT) [80,81]. WAT develops in all vertebrates, and invertebrates are also capable of storing neutral lipids in some organs. For instance, the midintestinal gland or hepatopancreas is a major lipid storage site in mollusks and crustaceans, and insects have “fat bodies” adjacent to the dorsal surface of the intestine [82,83,84]. In humans, WAT is an energy storage site and functions also as a passive element of the locomotory system [85]. The major sites of fat storage are the abdominal subcutaneous fat and the omental fat, and both can expand with overnutrition and cause obesity. Other white fat depots are relatively stable, such as the fat pads in the eye sockets or in the palmar surface of the hands or the plantar surface of the feet. In these cases, however, the fat pads function to reduce mechanical stress and to support the work of the muscles [85] (Figure 2A,B,G). Another relevant white fat depot is the female breast, which increases in size after puberty and particularly during pregnancy [86]. The white fat accumulated in the breasts supports the breast gland during lactation and, importantly, provides the necessary fat for the production of the breast milk [87].

All homeotherms depend on the maintenance of their core body temperature for survival, and BAT develops only in mammals, and has evolved to generate heat by increasing energy expenditure through actively oxidizing fatty acids and uncoupling mitochondrial respiration. The thermogenic activity of BAT is mediated by uncoupling protein 1 (UCP1), also termed thermogenin, which uncouples respiration from energy (ATP) generation. BAT was first described in the interscapular region of the hibernant marmot *(Marmota marmota,* basionym *“Mus alpinus”)* as early as 1551, and was denoted as “the hibernating gland” in the late 19th century [88]. In this sense, the brown adipose depot is among the longest studied metabolic organs. In rodents, BAT develops before birth, and has a metabolic role across the entire lifespan by supporting non-shivering thermogenesis [89,90,91]. UCP1-expressing, thermogenic fat cells may support core body temperature in recovery from hibernation in some mammals [88,92], and a cold environment also induces thermogenic fat expansion in humans [93]. Interestingly, several mammalian species that have adapted to a habitual cold environment lack UCP1 expression [94] or, alternatively, non-shivering heat generation occurs in the skeletal muscle without the need for thermogenic fat [95]. Similarly, human populations living in arctic environments do not develop excess thermogenic adipose tissue [96]. Thermogenic adipocytes are, hence, not the sole contributors to non-shivering thermogenesis, and the expansion of white adipocyte depots preserves core body temperature by offering an effective thermal insulation layer [88,97].

The ontogeny of the thermogenic fat cells is heterogenous: in mice, BAT shares progeny with the skeletal muscle, and brown adipocytes mostly develop from myogenic factor 5 (Myf5)-positive progenitors. Descendants of Myf5^+^ progenitors are scarce in WAT depots [98], and thermogenic adipocytes within the WAT develop independently from the Myf5^+^ lineage [98]. There is recent evidence showing that thermogenic fat cells can also develop from ectodermal progenitors, specifically from stem cells of the neural crest [99,100].

Recently, a new addition to the adipose tissue terminology has been introduced to describe thermogenic fat, namely “brite” (brown-in-white) adipose tissue, which is also known as induced brown adipose tissue or diffuse brown adipose tissue [90,101,102]. Brite adipose tissue was initially observed in the subcutaneous adipose tissue of rodents exposed to cold, and consists of both brown and white adipocytes. Beige adipocytes may develop without a cold stress, and are prevalent in some subcutaneous fat depots in the young [12,17] and in the adult mouse [102]. It is considered as a transitional form between white and brown adipose tissue, and its cells are often termed beige adipocytes [103]. Brown and beige adipocytes however have distinct ontogeny in the mouse [104], although they share some functional traits [17]. It has recently been suggested that adult humans have beige rather than brown adipocytes [105,106].

Thermogenic fat depots surround the kidney and arteries of vital organs throughout lifespan [75,76,77,107] (Figure 2A,B), and electron microscopy data show that the subcutaneous adipose tissue in the human newborn contains two types of cells. One type is large and has one large lipid droplet and small mitochondria, and the other is small and has several lipid droplets and contains large and numerous mitochondria [74] (Figure 2D). The subcutaneous adipose tissue of newborns expresses UCP1 and other gene products associated with brown adipocytes [12,17,56,68,69,74,108]. Similarly, the subcutaneous adipose tissue of the newborn mouse expresses gene products of mitobiogenesis and thermogenic fat differentiation [17]. In human infants, the level of UCP1 declines after six months of age, and is sensitive to the duration of breastfeeding [12,108].

Thermogenic (brown or beige) fat cells in the subcutaneous fat depot are present in children, and thermogenic potential is reactivated at puberty, concomitant with an increase in muscle mass [109]. During postnatal development, however, the majority of fat depots undergo a pronounced transformation that is usually accompanied by the loss of UCP1 [110]. Adipocytes in the human subcutaneous adipose tissue express UCP1 not only in infancy, but also in adulthood, and its level is positively correlated with that of *PRDM16* (PRD1-BF1-RIZ1 homologous domain containing 16), a transcriptional regulator and known inducer of thermogenic fat development [111]. The adult subcutaneous adipose tissue fat cell contains a much smaller number of mitochondria than equivalent newborn cells [56].

WAT (which predominates in the adult body) does not generally oxidize its fat stores, and ATP synthesis from the stored neutral lipids occurs only under periods of starvation and in response to exercise, when the fat cells hydrolyze the stored neutral lipids and produce fatty acids for the use of other metabolically active cells (Figure 2E). By contrast, the newborn mammal does not experience starvation as long as breastfeeding is provided [54]. Moreover, hormonal signals of lipolysis and uncoupling (i.e., β-adrenergic activation) are provided in the newborn [64]. Accordingly, the newborn adipose tissue is capable of actively oxidizing fatty acids to produce ATP and generate heat by mitochondrial uncoupling.

In summary, the human newborn has subcutaneous fat depots that contain both fat storing “white”, and fat oxidizing “brown” or “beige” adipocytes. This combination of fat storing ability and potential for lipolysis and burning-off lipids fulfils the dual metabolic role of the infant fat. However, the relative amount of mitochondria in these two cell types is different, allowing a changing degree of fat storage or fat catabolism. Obesity develops when fat catabolism is inhibited and lipogenesis is favored.

## 5. Mitochondria: Indispensable Organelles for Fat Catabolism

At the dawn of physiological chemistry in the middle 19th century, the German chemist Justus von Liebig (1803–1873) postulated that a negative correlation exists between the rate of respiration and obesity [112], albeit at the time neither the structure and molecular weight of lipids nor the biochemistry of fat oxidation was known [113]. Fat catabolism is indeed dependent on cellular respiration (that is, on the number and integrity of mitochondria). Mitochondria catalyze the β-oxidation of fatty acids and generate ATP from oxidative phosphorylation and heat from uncoupled oxidative phosphorylation. Carbohydrates can be used for ATP production without mitochondria owing to glycolysis. However, lipid breakdown requires mitochondria, and in some extent, peroxisomes. Neutral lipids and fatty acids are stored in the lipid droplet of adipocytes, and lipolysis occurs in the lipid droplet itself, but further processing of the released fatty acids requires mitochondria. When fat oxidation is intensive in fat cells, mitochondria are closely associated with lipid droplets, and the mitochondria may “engulf” the lipid droplets to allow its inner membrane to cover the lipid droplet [114]. Not surprisingly, brown and beige fat cells are rich in mitochondria, and actively mobilize lipids for energy and heat generation.

Due to the active lipolysis and fatty acid oxidation in adipocytes, the newborn fat reserves may be rapidly depleted if there is no sufficient supply from feeding. In a human infant, the heat generating fat exhausts its reserves within 36–48 h [70,115] and, accordingly, infections that cause diarrhea or excessive vomiting can lead to the loss of lipids from the thermogenic fat [72].

## 6. Breast Milk Signals Are Necessary for Mitochondrial Metabolism

**Figure 4:**
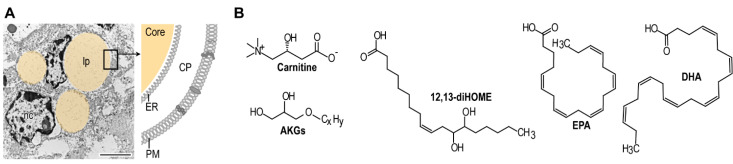
Breast milk lipids regulate fat metabolism. (**A**) Transmission electron microscopy image of a breast gland cell in lactating cattle. Lipid droplets are colored for better visibility. nc, nucleus; lp, lipid droplet (scale 2 μm). The scheme shows the morphology of the milk lipid droplets: it contains a triglyceride core (Core), which is surrounded by a single membrane layer of endoplasmic reticulum (ER), the cytoplasm (CP) and the plasma membrane (PM) of the gland cell. All of these compartments are rich in lipid species, and bear hallmarks of the maternal lipid profile [13], and transmit lipid signals from mother to child. For instance, breast milk ether lipids are used for synthesis of inflammatory lipid mediators in the neonate [119], which is believed to increase pathogen defense. Excess secretion of inflammatory lipid species into the breast milk can cause inflammatory disease in mice [120], and is believed to increase the development of chronic inflammatory diseases in human [41,121]. Breast milk lipid species are also responsible for proper adipose tissue development and functioning in the infant [12,19]. (**B**) Lipid species of breast milk that shape the development and function of adipocytes. AKGs, alkylglycerols; 12,13-diHOME, 12,13-dihydroxy-9Z-octadecenoic acid; EPA, eicosapentaenoic acid (ω-3 fatty acid); DHA, docosahexaenoic acid (ω-3 fatty acid).

Importantly, breast milk lipids are not only metabolic fuel, but also function as signal transmitters, and the type of postnatal feeding affects metabolism [116] and adipose tissue development in the newborn [12] (Figure 4). For instance, breast milk contains carnitine, which is necessary for the transport of fatty acids into the mitochondrial stroma for subsequent β-oxidation and energy production [117]. Breast milk carnitine thus supports mitochondrial fatty acid oxidation in the infant. Breast milk also contains long-chain polyunsaturated fatty acids (PUFAs), such as docosahexaenoic and arachidonic acid. PUFAs incorporate into the mitochondrial membranes, have complex roles in mitochondrial free radical production and the initiation of the mitochondrial pathway of apoptosis, and their metabolites induce the gene transcription of mitochondrial enzymes, including components of the β-oxidation [4,118].

Breast milk of mothers with overweight and obesity have higher ω-6 PUFAs levels and lower ω-3 PUFAs levels than the breast milk of normal-weight mothers [122], and it has been shown that the ratio of ω-6 relative to ω-3 (ω-6/ω-3) fatty acids in human breast milk has increased three-fold over the last 30 years [123]. Dietary intake of ω-3 PUFAs increases thermogenic fat development in adulthood [124,125], and an increased ω-6/ω-3 ratio of fatty acids in the colostrum is associated with increased adiposity in the first 6 months of life [122]. However, breast milk ω-3 PUFAs (unlike colostrum ω-3 PUFAs) do not affect [126] or may even increase fat mass in the first year of life [127], probably due to the fact that ω-3 PUFAs may be used for de novo lipid synthesis instead of fatty acid oxidation [128]. PUFAs hence may have distinct effects immediately after birth (when colostrum is consumed and a rapid lipolysis appears) and later in infancy (when breast milk is consumed and lipogenesis is ongoing). PUFAs can be metabolized into lipid mediators, or can act through free fatty acid receptor 4 (FFAR4, also termed GPR120).GPR120 deficiency causes obesity in mouse and human, due to increased adipocyte differentiation and lipogenesis in the liver [129]. However, adipose tissue of the infant mouse, which is rich in thermogenic adipocytes, expresses lower levels of *Ffar4* than its adult counterpart [17].This may explain the distinct effects of PUFAs on thermogenic fat development in infancy and in adulthood. PUFAs also have effects that are independent from GPR120 signaling, since they are precursors of lipid mediators, such as oxylipins, eicosanoids and thromboxanes, which have physiological role in the newborn (reviewed in [130]). These lipid mediators are also present in the breast milk [131], and some oxylipins are associated with BAT in mouse [132].

The BAT-derived lipid metabolite 12,13-dihydroxy-9Z-octadecenoic acid (12,13-diHOME), also known as isoleukotoxin, is a circulating lipokine released by brown adipocytes in response to exercise and cold exposure [133], and is also present in human breast milk [19]. Greater breast milk abundance of 12,13-diHOME at one-month postpartum is associated with lower subcutaneous fat mass in the infant, and a reduced gain in body mass index in the first six months of infancy [19]. Thus, 12,13-diHOME and its related metabolites appear to protect against adiposity in infancy, which is similar to what has been reported in adults [134].

Carnitine, PUFAs and 12,13-diHOME are supplied not only in breast milk, but also in commercially available infant formula. Moreover, 12,13-diHOME is produced by the gut microbiota and can be found on the surface of neonatal skin [19]. Other breast milk-specific lipid species, the so-called alkylglycerol-type ether lipids (AKGs), are not detectable in commercially available cow milk-based infant formula [19]. AKGs are metabolized by resident macrophages of the infant adipose tissue to platelet-activating factor (PAF) and related lipid transmitters [12]. The adipose tissue of the young mouse shows elevated PAF-mediated signaling, and compromised PAF signaling triggers obesity [12,17]. AKG-mediated signaling triggers mitobiogenesis and thermogenic fat development in the infant, thereby supporting the catabolism of dietary lipids [12]. AKGs are synthesized during PAF remodeling [135]. AKGs are membrane lipids present in Archaea, and the level of methanogenic Archaea-derived metabolites correlate with the stage of lactation in cattle [136]. This suggests that breast milk AKGs may have their origin in the human microbiota, although this possibility has not been tested. AKGs have some structural similarity to pristane and phytane, which are chlorophyll metabolites. Pristane and phytane are present in human adipose tissue [137], and they are incorporated into adipose tissue in rat [138]. A related metabolite, phytol, is known to induce thermogenic fat development in the mouse [139]. Thus, AKGs and their metabolites are inducers of thermogenic fat development, and are maternal signals of infant-type fat development.

An intriguing issue is the lack of AKGs in cattle milk, which contrasts with their presence in the milk of humans and many mammalian species. Modern milk-producing cattle breeds are descendants of a small number of founders, approximately as few as 80 animals, that were domesticated from wild ox in the Near East approximately 10,500 years ago [140]. The wild ox (*Bos taurus primigenius†*) survived in Europe until 1627, when the last recorded aurochs died in the Jaktorôw Forest in Poland [141], making it impossible to assess AKG content of the wild ox breast milk. The founder effect and the high degree of endogamy raise the possibility of genetic drift that has led to the loss of AKG production in domesticated cattle. Moreover, the high volume of produced milk may lead to the dilution of AKGs in cattle milk; alternatively, the presence of AKG-degrading enzymes in cattle milk might explain their absence. As infant formula is more often than not cattle milk-based, AKGs are lacking from infant formulas [142].

It has been shown that the lack of AKG intake in newborn mice leads to premature loss of thermogenic fat cells in the subcutaneous adipose tissue, concomitant with obesity [12,31,35]. Similarly, insufficient breastfeeding promotes childhood obesity and increases the risk of inflammatory diseases and diabetes later in life [31,35]. This may be due to the lack of specific breast milk lipid signals, or to the presence of certain microRNA species or endotoxin in the infant formula, which serve as lipogenic signals and promote obesity [12,143,144] (Figure 3). Breast milk lipids hence constitute a unique molecular axis of communication between the mother and the child, and are powerful determinants of adipose tissue development.

## 7. Evolution and Current Impact of Breastfeeding

**Figure 5:**
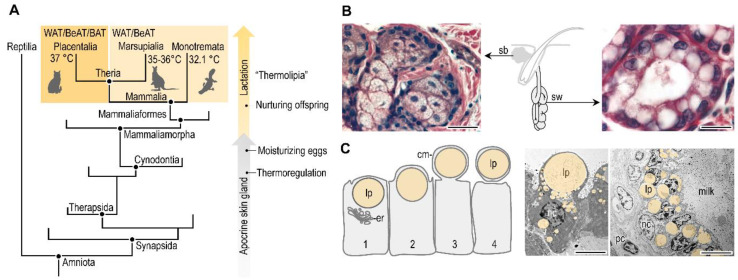
Evolution of breastfeeding. (**A**) Simplified phylogenic tree of mammals and their evolutionary forerunners. Relative time scale of the acquisition of homeothermy and the advent of lactation are shown. Placentalia have a core body temperature of 37 °C, and develop white adipose tissue (WAT) and thermogenic adipose tissue: brown adipose tissue (BAT) and beige- or induced brown adipose tissue (BeAT). Marsupialia have a lower core body temperature (35–36 °C) than the mammals, such as the Monotremata (around 32 °C). Marsupialia and Monontremata develop WAT, while BeAT only upon induction [160,161]. Developing BeAT depots is a form of adaptive thermogenesis. (**B**) Histology of the mammalian skin glands: sebaceous gland (sb) and sweat gland (sw) [162]. The sebaceous gland cells are filled with lipid droplets and undergo apoptosis as a mechanism of holocrine lipid secretion. The sweat glands have an apocrine secretion mechanism, and the image shows large secretory droplets in the apical regions of the gland cells. Hematoxylin and eosin staining, scale 25 μm. Both glands are associated with the hair follicle. (**C**) Lipid secretion mechanism of the lacteal gland (or breast gland) [12]. Note the apocrine release of the lipid droplet (lp), enveloped in the cell membrane (cm). er: endoplasmic reticulum. Transmission electron microscopy images of gland cells in the breast of lactating mouse and cattle. nc: nucleus, lp: lipid droplet, pc: plasma cell. Scale bar 2 μm.

Lactation and a fatty acid-based metabolism, along with the presence of fat oxidizing or thermogenic fat in the newborn, are reproductive traits of extant mammals (Figure 5A). This suggests a shared evolutionary history of the two traits, and a possible link between breastfeeding and thermogenic fat differentiation in the breastfed infant. Both breastfeeding and thermogenic potential of the adipose tissue have evolved in the quest for maintaining stable core body temperature. The mammary gland, also called the lacteal gland, has evolved from sweat glands in the so-called Synapsid clade (Figure 5A). Sweat glands are cutaneous glands that control body temperature by evaporating a watery secretion on the skin surface (Figure 5B), and having sweat glands is a step towards active regulation of body temperature. The Synapsid clade has evolved from stem amniotes, and diverged from Diapsids, the line of the todays living reptiles and birds. It is thought that extinct non-mammaliaform synapsid species, also called proto-mammalians, had sweat glands associated with hair follicles [145]. The non-mammalian synapsids laid eggs that had a thin, leathery shell vulnerable to desiccation. Hence, it is thought that an additional function of the secreted liquid was to moisturize the eggs and foster the offspring. The extant monotremes also lay eggs, and have lacteal glands that develop from apocrine sweat glands associated with hair follicles, secreting milk onto the surface of the skin [146,147]. It is plausible that the egg-moisturizing glands of the non-mammalian synapsids are evolutionary forerunners of the lacteal glands, and ultimately the mammary gland of mammals. Accordingly, lactation might have evolved in some non-mammalian synapsids before the origin of mammals, about 240 to 246 million years ago, along with the advent of homeothermy [145].

The mammary gland of mammals is a specialized form of hair follicle-associated apocrine sweat gland (Figure 5B,C). The development of the hair follicles and the mammary gland in mammals is controlled by the same homeobox transcription factors, including Msx1 and Msx2 [148]. Moreover, the lacteal glands of the monotremes secrete milk into the hair follicles. This is strong evidence of the shared evolutionary history of sweat glands and the mammary gland, and suggests that lactation has evolved from a body temperature-regulating organ, i.e., the sweat gland.

Breastfeeding seems to control another trait of homeothermy, the ability of heat production in the infant. After birth, the newborn enters a thermally-challenging environment, and most of the energy metabolism supports the maintenance of body temperature [62]. This needs not only high-energy metabolic fuels such as lipids, but also lipid mediators (e.g., carnitine, AKGs, 12,13-diHOME), which support the development of the mitochondrial network in the adipocytes, may aid the mitochondrial uncoupling, eventually, heat production in the adipose tissue. Placental mammals have well-defined thermogenic adipose tissue depots, while marsupials and monotremes develop thermogenic fat only in response to specific stimuli (Figure 5A). Fatty acids involved in lipogenesis are rich in the monotreme milk [149], and fat of the platypus milk contains 98.5% triglycerides [150]. In marsupials the milk lipid content increases gradually during early lactation, and increases exponentially in late lactation and reaches a peak around weaning. This increase of milk lipid content helps to build up energy reserves for thermoregulation (reviewed in [151]).

Lactation is essential for postnatal development in all mammals. Today, however, humans have intentionally reduced breastfeeding duration, and instead make extensive use of formula milk. Industrialization, social and cultural impact are the most likely reasons for declining breastfeeding rates and low-income societies have a preference of premature weaning of infants [63,152,153]. Breastfeeding rates continue to decline worldwide and 60% of infants are currently never breastfed [35]. For instance, the COVID-19 pandemic in 2020–2021 has further increased the formula-feeding rate, due to the fear of mother-to-child infection through breast milk [152].

Today at least six months of exclusive breastfeeding is a WHO recommendation for newborns [35]. However, archeological evidence of skeletal remains from the Bronze Age (ca. 2800–1200 BCE), Roman age and Medieval times (700 BCE–AD 1500) suggest that exclusive breastfeeding spanned six months followed by a gradual introduction of weaning food, and the actual weaning age was between two to four years depending on culture and location [154,155]. In Early and Late Neolithic groups, breast milk was the major protein source until the age of two to three years; however, there are differences in the age of weaning completion and duration: Early Neolithic groups weaned their infants at a later age and over a shorter period of time [156]. Agriculture has shortened the duration of breastfeeding, allowing longer recovery periods between pregnancies and allowing expansion of the population. Social status may have also had an impact on infant feeding decisions [157]. This information has been obtained by d^15^N isotope analysis of skeletal remains, the so-called weaning age reconstruction with nitrogen isotope analysis. In brief, the trophic level of the mother and the child are different, and breastfed infants have a higher degree of ^15^N incorporation from breast milk than weaned children who have a lower ^15^N isotope level with age [154]. It is thought that breastfeeding length was even longer, up to four to five years before the advent of agriculture in the Neolithic period. In the Medieval period, weaning age was around two years of age, or later. For instance, in medieval Japan the dietary protein contribution from breast milk started to decrease from 1.1 years of age and ended at 3.8 years [158]. In Shakespeare’s Romeo and Juliet, one of the lengthy monologues of the nurse reveals that Juliet was weaned at the age of six [159].

Overall, exclusive breastfeeding and partial breastfeeding provided an important part of infant nutrition in the past. As a comparison, exclusive breastfeeding is today limited to a few days or weeks, and most infants receive formula milk before the age of three months, with ~5% of infants worldwide never breastfed [42]. The industrial revolution has brought on this dramatic decline of breastfeeding, and can be attributed to societal changes and to some health reasons. One is the fear from rickets and the transmission of diseases with breast milk, such as syphilis [63]. Rickets can be prevented and/or treated with calcium and vitamin D supplementation, and since breast milk is low in vitamin D the use of a fortified baby formula seemed an attractive solution [13]. However, the fetal adipose tissue builds up vitamin D stores before birth [163], and rickets is a modern era disease, and skeletal remains with bone abnormalities associated with rickets are rare in the Neolithic and Medieval times when infants were breastfed for a much longer duration than today [154].

Paradoxically, unlike other mammals, humans consume breast milk of other mammals in form of dairy products in adulthood. This is a specific trait of human nutrition, and it is thought to have its roots in the Neolithic. Post-glacial Europe has archeological artefacts of pottery to store and process milk, most likely to produce cheese [164]. The majority of adult humans lack the ability to digest lactose. However, cheese and other fermented milk products have lower lactose levels than milk, and hence fermented dairy products are widespread in human nutrition to increase the intake of protein, calcium, and vitamin D [164]. However, it is plausible that animal milk has non-favorable effects in the newborn [143,144,165,166], and we still know little about the mechanism(s) of action of breast milk-derived lipid mediators that support the metabolism of fat in the newborn.

## 8. Conclusions

The human newborn utilizes lipids to maintain metabolic homeostasis, and breast milk provides lipid mediators to support the development and function of adipocyte mitochondria. Mother-to-child signaling through breast milk shapes infant fat metabolism, and its impairment may be an early life trigger of obesity. The underlying mechanisms, such as the biosynthesis of breast milk lipid mediators, and the signal mechanisms they activate in the infant, are only now beginning to be explored. Intriguingly, there are sex-specific differences in the composition of breast milk [167], and boys and girls show different fat development and fat distribution [168], making it conceivable that fat-regulating signals may also have sex-specific differences in the breast milk. Insufficient breastfeeding and premature weaning are potential triggers of obesity and various immune-metabolic alterations, including diabetes and self-immunity, highlighting the importance of increasing the rates of breastfeeding [12,49].

## Data Availability

Not applicable.
